# Burn epidemiology - an Indian perspective

**Published:** 2009

**Authors:** Sameek Bhattacharya

**Affiliations:** Department of Plastic & Reconstructive Surgery, PGIMER, R.M.L. Hospital, New Delhi, India

Epidemiological study is an important modality to analyze the cause, magnitude and profile of burn in a particular region and population. Epidemiological study is the first step in planning preventive and management strategies; hence, any endeavor in this direction is appreciable.

Burn profile closely follows the socioeconomic flux of a country.[1] Economically developed nations with sound prevention policy, organized dwelling and safe kitchen technology and fuel have brought down burn incidence drastically. However, in developing nations, burn continues to be endemic because of massive slum dwelling and large scale use of unsafe stoves and fuel.

A recent study by Ahuja et al,[2] documents that economic uplift and shift from kerosene to safer LPG stoves has brought down annual burn admission by 43% in a major burn unit of Delhi.

The decrease of burn incidence in the developed world is mainly in the adult population. Children continue to be vulnerable to burns in nuclear families with both parents working. On the other hand, in developing countries like India and Africa the young adult population engaged in the kitchen gets burnt more frequently compared to children. The major difference in the pediatric burn profile of developed and developing nations is that pediatric burn in the former is mainly scalds, whereas, in the latter a large number of children sustain flame burn. This can be attributed to congested living and floor level cooking. This is evident in the present study as well as many reports from India and other developing countries.[3–6].

This study does not mention overall burn admission and the number of adult burns, however, considering the socioeconomic scenario of Nigeria it is quite probable that burn incidence will be very high and adult burns far more prevalent as is the case in India. The author acknowledges this possibility of higher burn incidence and of many patients being treated elsewhere. In future it will be more worthwhile to report a multi-centric data of the overall burn epidemiological scenario. It will go a long way in devising preventive and management strategies.

## References

[1] Forjuoh SN (2006). Burns in low and middle income countries: A review of available literature on descriptive epidemiology, risk factors, treatment and prevention. Burns.

[2] Ahuja RB, Bhattacharya S, Rai A (2009). Changing trends of an endemic trauma. Burns.

[3] Ahuja RB, Bhattacharya S (2002). An analysis of 11,196 burn admissions and evaluation of conservative management techniques. Burns.

[4] Jayaraman V, Ramakrishnan KM, Devies MR (1993). Burns in Madras: An analysis of1368 patients in one year. Burns.

[5] Subrahmanyam M (1996). Epidemiology of burns in a district hospital in western India. Burns.

[6] Laloe V (2002). Epidemiology and mortality of burns in a general hospital of Eastern Sri Lanka. Burns.

[7] Oludiran OO, Umebese PF (2009). Pattern and outcome of children admitted for burns in Benin city, mid-western Nigeria. Indian J Plast Surg.

